# Single Nucleotide Polymorphisms (SNPs) of *RAD51*-G172T and *XRCC2*-41657C/T Homologous Recombination Repair Genes and the Risk of Triple- Negative Breast Cancer in Polish Women

**DOI:** 10.1007/s12253-015-9922-y

**Published:** 2015-03-07

**Authors:** Magdalena M. Michalska, Dariusz Samulak, Hanna Romanowicz, Beata Smolarz

**Affiliations:** 1 Department of Obstetrics and Gynaecology, Regional Hospital in Kalisz, Kalisz, Poland; 2 Cathedral of Mother’s and Child’s Health, Poznan University of Medical Sciences, Poznań, Poland; 3 High School of Informatics, Regional Hospital in Glowno, Glowno, Poland; 4 Laboratory of Molecular Genetics, Department of Fetal-Maternal Medicine and Gynecology, Institute of Polish Mother’s Memorial Hospital, Lodz, Poland, Rzgowska 281/289, 93-338 Lodz, Poland

**Keywords:** *RAD51*, *XRCC2*, Triple negative breast cancer, Gene polymorphism

## Abstract

Double strand DNA breaks are the most dangerous DNA damage which, if non-repaired or misrepaired, may result in genomic instability, cancer transformation or cell death. *RAD51* and *XRCC2* encode proteins that are important for the repair of double-strand DNA breaks by homologous recombination. Therefore, genetic variability in these genes may contribute to the occurrence and progression of triple-negative breast cancer. The polymorphisms of the *XRCC2* gene -41657C/T (rs718282) and of the *RAD51* gene, −172G/T (rs1801321), were investigated by PCR-RFLP in 70 patients with triple-negative breast cancer and 70 age- and sex matched non-cancer controls. The obtained results demonstrated a significant positive association between the *RAD51* T/T genotype and TNBC, with an adjusted odds ratio (OR) of 4.94 (*p* = 0.001). The homozygous T/T genotype was found in 60 % of TNBC cases and in 14 % of the used controls. Variant 172 T allele of *RAD51* increased cancer risk (OR = 2.81 (1.72–4.58), *p* < .0001). No significant associations were observed between -41657C/T genotype of *XRCC2* and the incidence of TNBC. There were no significant differences between the distribution of *XRCC2* -41657C/T genotypes in the subgroups assigned to histological grades. The obtained results indicate that the polymorphism of *RAD51,* but not of *XRCC2* gene, may be positively associated with the incidence of triple-negative breast carcinoma in the population of Polish women.

## Introduction

Triple-negative breast cancer (TNBC) is a subtype of breast cancer defined by the lack of expression of estrogen, progesterone and HER-2 (human epidermal receptor-2) receptors. TNBC refers to about 15–20 % of all breast cancer cases. It is characterised by worse clinical outcome, poor prognosis and absence of prognostic indicators [1–5].

Mutations in DNA of double-strand breaks (DSB) repair genes are involved in the pathogenesis of tumours, however, it is still unclear whether any defects in this pathway may play any role in the aetiology of triple-negative breast cancer.

DSB in DNA may be rectified either by homologous recombination (HR) or non-homologous end joining (NHEJ) [6, 7].

RAD51 is involved in the homologous recombination and repair of double-strand breaks in DNA and DNA cross-links and is responsible for the maintenance of chromosome stability [8].

*RAD51* gene has been mapped to chromosome 15q14-15 and is polymorphic in nature. The involvement of *RAD51* in the DNA repair determines its potential role in maintaining genetic stability, which is disturbed in cancer. Therefore the problem of genetic variability of the *RAD51* gene in cancer is worthy studying. A substitution G/T at position 172 in 5′ untranslated region of the *RAD51* gene (the 172G/T 5′UTR polymorphism) has been reported as a possible factor to affect the *RAD51* mRNA stability [8, 9]. 172G/T polymorphism of *RAD51* is located in the regulatory element of the *RAD51* promoter and is suggested to be associated with messenger RNA expression [8, 9].

In literature, many of researches suggest that 5′UTR polymorphism of *RAD51* gene may contribute to mammary carcinogenesis. However, the reported results have rather been inconsistent [10–16].

*XRCC2* gene, located in 7q36.1, is an essential part of the homologous recombination repair pathway and a functional candidate for involvement in cancer progression [17].

It is known that polymorphisms in *XRCC2* may modify individual susceptibility to breast cancers [13, 14, 18–20]. Common variants within *XRCC2*, particularly a coding single nucleotide polymorphism (SNP) in exon 3 (Arg188His, R188H, rs3218536), have in recent studies been identified may be associated with a significantly increased risk of breast cancer [13, 14, 18–20].

Unfortunately, it is difficult to find in the literature reports directly binding **-**41657C/T SNP in DNA repair gene *XRCC2* with breast cancer occurrence.

Because little is known on the association between *RAD51*-172G/T and *XRCC2*-41657C/T polymorphisms and TNBC, it was studied whether the polymorphisms in the two genes, being involved in the homologous recombination of double-strand breaks, could have been linked with the risk for triple-negative breast cancer in that Polish population.

## Materials and Methods

### Patients with Triple- Negative Breast Cancer

A total of 70 patients with histologically-proven diagnosis of TNBC were included in the reported study. The distributions of sociodemographic characteristics, lifestyle risk factors and clinical characteristics of the patients are shown in Tables 1 and 2, respectively. Paraffin embedded tumour tissue specimens were obtained from women with triple-negative breast carcinoma, treated at the Department of Oncology, Institute of Polish Mothers Memorial Hospital, between 2000–2013. The age of the patients ranged in from 36 to 68 years (the mean age 46.2 ± 10.12). The median follow-up of patients at the time of analysis was 38 months (the range: 2–70 months). The average tumor size was 20 mm (the range 17–32 mm). All the diagnosed tumours were graded by criteria of the Scarf-Bloom-Richardson. DNA from non-cancerous breast tissue (*n* = 70) served as control (mean age 45.41 ± 18.21). The Local Ethical Committee approved the study and each patient gave a written consent for participation in the study.

**Table 1:** Characteristics of breast cancer patients^a^ with questionnaire data

Characteristics	Number of cases (%)
Age (years)	
< 45	27 (39)
45–54	15 (21)
55–64	18 (26)
> 64	10 (14)
Family history of breast cancer^b^	
Yes	25 (36)
No	45 (64)
BMI (body mass index) (kg/m^2^)	
< 24.9	25 (36)
25–29.9	20 (28)
> 30	25 (36)
Smoking status^c^	
Never	23 (33)
Ever	27 (39)
Moderate	10 (14)
Heavy	10 (14)
Alcohol intake^d^	
Never/rare	22 (31)
Light	18 (26)
Moderate	10 (14)
Heavy	9 (13)
Ex–drinker	11 (16)
Menarche (years)	
10	5 (7)
11	18 (26)
12	19 (27)
13	13 (19)
14	10 (14)
≥ 15	5 (7)
Parity	
Nulliparous	25 (36)
1	15 (21)
2	16 (23)
3	
≥ 4	9 (13)
5 (7)	
Menopause status	
Premenopausal	30 (43)
Postmenopausal	40 (57)
Use of menopausal hormones^e^	
Never	37 (53)
Estrogen	33 (57)

^a^ *n* = 70

^b^Family history defined as self-reporting of at least one first-degree relative with known breast cancer

^c^Non-smoking patients (never), patients smoking 10 cigarettes per day for 10 years (ever), patients smoking 20 cigarettes per day for 20 years (moderate) and patients smoking 20 cigarettes per day for thirty years (heavy)

^d^Never/rare,<1 U/week; light, 1–8.9 U/week; moderate, 9–17.9 U/week; heavy, ≥18 U/week; where 1 U = 22 g ethanol

**Table 2:** Characteristic features of triple-negative breast cancer patients^a^

Triple-negative breast cancer	Patients (*n* = 70)	
	n	%
Scarf-Bloom-Richardson stage		
I	20	29
II	45	64
III	5	7
Tumor size grade		
T1	8	11
T2	40	57
T3	18	26
T4	4	6
Lymph node status		
N0	32	46
N1	12	17
N2	14	20
N3	7	10
N4	5	7

^a^ *n* = 70

Breast tissue samples (cancerous and non-cancerous) were routinely fixed in phormaldehyde, embedded in paraffin, cut into thin slices and stained with haematoxylin/eozin for pathological examination. DNA for analysis was obtained from archival pathological paraffin-embedded tumour specimens and from samples of non-cancerous breast tissue, which were deparaffinised in xylene and rehydratated in ethanol and distilled water. In order to ensure that the chosen histological material was representative for cancerous and non-cancerous tissue, each tissue sample, qualified for DNA extraction, was initially checked by a pathologist. DNA was extracted from the material, using a commercially available QIAmp DNA purification kit (Qiagen GmbH, Hilden, Germany), according to the manufacturer’s instruction.

### Genotype Determination

The PCR- restriction fragment length polymorphism method (PCR-RFLP) was used to detect the genotypes of the -41657C/T and -172G/T polymorphisms as described previously [21–23].

### Statistical Analysis

For each polymorphism, departure of the genotype distribution from that expected from Hardy-Weinberg equilibrium was assessed using the standard *χ* ^2^-test. Genotype frequencies in the study cases and the controls were compared by *χ* ^2^-test. Genotype specific risks were estimated as odds ratios (ORs) with associated 95 % intervals (CIs) by unconditional logistic regression. *P*-values < 0.05 were considered significant. All the statistical analyses were performed, using the STATISTICA 6.0 software (Statsoft, Tulsa, Oklahoma, USA).

## Results

Table **3** shows genotype distribution of *RAD51* polymorphism, illustrating the difference between the patients with TNBC and the controls. The Table clearly indicates significant differences (*p* < 0.05) between the two investigated groups. A weak association was observed between the occurrence of triple-negative breast carcinoma and the presence of G/T and T/T genotypes. A stronger association was observed for T/T homozygotes than for G/T heterozygotes. Variant 172 T allele of *RAD51* increased cancer risk. The frequencies of G/G, G/T and T/T genotypes, observed in the study patients, differed significantly (*p* < 0.05) from the distribution values, as expected from the Hardy-Weinberg equilibrium.

**Table 3:** Distribution of *RAD51* genotype and allele frequencies in patients with triple- negative breast cancer and in control groups

	Patients with TNBC *n* = 70		Controls *n* = 70			
172G/T	Number	(%)	Number	(%)	OR (95 % CI)^a^	*p* ^b^
G/G	17	24	20	29	1.00 Ref	
G/T	11	16	40	57	**0.32 (0.13–0.82)**	**0.028**
T/T	42	60	10	14	**4.94 (1.91–12.71)**	**0.001**
G	45	32	80	57	1.00 Ref	
T	95	68	60	43	**2.81 (1.72–4.58)**	**<.0001**

Data in bold font are statistically significant

^a^Crude odds ratio (OR), 95 % CI = confidence interval at 95 %

^b^Chi square

No statistically significant differences were observed between the control group and the patients with triple-negative breast cancer, regarding either allele or genotype frequencies of *XRCC2* -41657C/T gene polymorphism (see Table 4).

**Table 4:** Distribution of *XRCC2* genotype and allele frequencies in patients with triple- negative breast cancer and in control groups

	Patients with TNBC *n* = 70		Controls *n* = 70			
−41657C/T	Number	(%)	Number	(%)	OR (95 % CI)^c^	*p* ^b^
C/C	18	26	17	24	1.00 Ref	
C/T	35	50	37	53	0.89 (0.39–2.00)	1.000
T/T	17	24	16	23	1.00 (0.38–2.59)	0.806
C	71	51	71	51	1.00 Ref	
T	69	49	69	49	1.00 (0.62–1.59)	0.920

^a^Crude odds ratio (OR), 95 % *CI* confidence interval at 95 %

^b^Chi square

Scarf-Bloom-Richardson grading was related to *RAD51* and *XRCC2* polymorphisms. Histological grade was evaluated in all the cases (*n* = 70), where 20 cases were of grade I, 45 of grade II and 5 of grade III. Grade II and III were grouped together for the purposes of statistical analysis (see Table 5). No differences were observed in those groups between *RAD51* and *XRCC2* genotype distributions, while a lack of correlation was demonstrated between genotypes of the identified polymorphisms and TNBC invasiveness.

**Table 5:** Dependence of *RAD51* and *XRCC2* gene polymorphism genotypes and allele frequency on tumour grade in patients with triple–negative breast cancer^a^

grade^b^	I (*n* = 20)	II + III (*n* = 50)	OR (95 % CI)^c^	*p* ^d^
*RAD51* 172G/T	Number (%)	Number (%)		
G/G	4 (20 %)	13 (26 %)	1.00 Ref	
G/C	2 (10 %)	9 (18 %)	0.72 (0.10–4.82)	0.560
C/C	14 (70 %)	28 (56 %)	1.62 (0.44–5.91)	0.671
G	10 (25 %)	35 (35 %)	1.00 Ref	
C	30 (75 %)	65 (65 %)	1.61 (0.70–3.68)	0.345
*XRCC2* 41657C/T				
C/C	4 (20 %)	14 (28 %)	1.00 Ref	
C/T	14 (70 %)	21 (42 %)	1.19 (0.33–4.23)	0.526
T/T	2 (10 %)	15 (30 %)	0.47 (0.07–2.95)	0.357
C	22 (55 %)	49 (49 %)	1.00 Ref	
T	18 (45 %)	51 (51 %)	0.79 (0.37–1.64)	0.647

^a^ *n* = 70

^b^according to Scarf-Bloom-Richardson criteria

^c^Crude odds ratio (OR), 95 % *CI* confidence interval at 95 %

^d^Chi square

We did not find any association of the *XRCC2* and *RAD51* polymorphisms in patients group with cancer progression assessed by breast cancer with (N+) and without (N-) lymph node metastases (*p* > 0.05). Our data did not demonstrate any statistically significant correlation between *RAD51* and *XRCC2* polymorphisms and the risk factors for breast cancer, such as BMI (body mass index), smoking status, alcohol consumption, menarche, menopause status, reproductive histories, exogenous hormone use and medical history (data not shown).

## Discussion

Breast cancer classification is in constant evolution, as advances in molecular pathology as well as immunohistochemical staining allow researchers to define the molecular heterogenity of different disease subtypes and to guide the selection of appropriate treatment. The triple negative phenotype, defined as the lack of estrogen receptor, progesterone receptor and HER-2 expression, represents approximately 15-20 % of breast cancer cases and has a worse clinical outcome and prognosis than other breast cancer subtypes [1–5].

As it has already been mentioned above, little is known on the association between the homologous recombination repair *RAD51* and *XRCC2* SNPs and triple-negative breast cancer. Therefore, it was attempted to investigate the role of genetic variation in homologous recombination repair genes and the risk of TNBC.

As mentioned in Introduction mutations in DNA double-strand breaks repair genes are involved in the pathogenesis of tumours. Defects in this pathway may play a role in development and progression of breast cancer [24, 25].

In the literature, many reports confirm the significance *RAD51* gene polymorphisms in the 5′UTR, regarding the risk of breast carcinoma [16, 26–28]. However, the reported results have rather been inconsistent [10–16].

No significant associations were observed between the 172G/T and breast cancer in Korean women. The genotype distributions of *RAD51* SNP did not differ significantly from Hardy-Weinberg equilibrium (*P* > 0.05) and the frequencies of minor alleles were 0.13 for *RAD51* 135C and 0.05 for *RAD51* 172 T. When the two loci in the same gene were evaluated for linkage disequilibrium, two loci in *RAD51* gene (135G/C and 171G/T) were in negative linkage disequilibrium [15].

Shin et al. examined the role of SNPs in *RAD51* repair genes and the risk of breast cancer. In the reported study, the 135G/C but not 172G/T polymorphism, of *RAD51* gene, was associated with breast carcinoma occurrence [16].

Other experimental study confirms the significance *RAD51* gene G135C polymorphism, regarding the risk of breast carcinoma [10–14].

The role of -41657C/T variation of *XRCC2* in breast cancer is still unknown. Some reports documented that the *XRCC2* -41657C/T genotype was related to increased esophageal squamous cell carcinoma (ESCC), gastric cardia adenocarcinoma (GCA) and in smoking- drinking-related laryngeal cancer risk [22, 29].

It appears from literature review that *RAD51* and *XRCC2* polymorphisms have been investigated in various types of malignancy. A large number of studies confirm a significant role of the influence of specific genetic variants on repair phenotype and cancer risk [29–34].

Unfortunately, it is difficult to find reports in the available literature, which would directly bind *RAD51* and *XRCC2* SNP in DNA repair gene by HR with clinical-pathological features of breast tumour. Only in single studies the researchers suggest that the homologous recombination repair gene polymorphism may play some role in the development of breast carcinoma [13].

Two single nucleotide polymorphisms of *XRCC2*-41657C/T, and *RAD51*-172G/T were investigated in the reported study, as well as their association with human triple- negative breast cancer.

In literature, neither *XRCC2*-41657C/T nor *RAD51*-172G/T polymorphism is correlated with risk of breast cancer [15, 16]. What are important, recent reports introduce the role of *RAD51* 135G/C polymorphism in the development of triple-negative breast cancer [27].

In the recent studies, 135G/C polymorphism of *RAD51* may be associated with an elevated tumour risk in the Polish populations, regarding TNBC [28], while there are still no data, which would be illustrating the significance of *RAD51* polymorphism for triple-negative breast cancer development in other populations.

The obtained results confirm the important role of *RAD51* G172T polymorphism for TNBC occurrence in Poland. In the reported study, *RAD51* T/T genotype increased the risk of breast cancer in the Polish population. There was a 4.94-fold increased risk of breast carcinoma for *RAD51*-T/T genotype carriers, compared with subjects with *RAD51*-G/G, G/T genotype. The 172 T allele increased the risk of TNBC. *RAD51* G172T polymorphism was not related to cancer grade. It is possible that the presence of the 172 T allele is in some disequilibrium with another, so far unknown, mutation, located outside the coding region in *RAD51* gene, which may be of importance for RAD51 concentration in plasma.

No significant associations were observed between -41657C/T genotype of *XRCC2* and the incidence of TNBC in the Polish women.

In conclusion, the obtained data confirm the important role of *RAD51* 172G/T - but not of -41657C/T of *XRCC2* gene polymorphism in triple-negative breast cancer aetiology.

Finally, we suggest that *RAD51* 172G/T may be used as a prognosing factor of precancerous lesions, predicting TNBC in the Polish population. Further studies are required, regarding the role of these genes in the aetiology of triple-negative breast cancer.

## References

[1] Bauer KR, Brown M, Cress RD, Parise CA, Caggiano V (2007). Descriptive analysis of estrogen receptor (ER)-negative, progesterone receptor (PR)-negative, and HER2-negative invasive breast cancer, the socalled triple-negative phenotype: a population-based study from the California Cancer Registry. Cancer.

[2] Chacón RD, Costanzo MV (2010). Triple-negative breast cancer. Breast Cancer Res.

[3] Cleator S, Heller W, Coombes RC (2007). Triple-negative breast cancer: therapeutic options. Lancet Oncol.

[4] Dawson SJ, Provenzano E, Caldas C (2009). Triple negative breast cancers: clinical and prognostic implications. Eur J Cancer.

[5] de Ruijter TC, Veeck J, de Hoon JP, van Engeland M, Tjan-Heijnen VC (2011). Characteristics of triple-negative breast cancer. J Cancer Res Clin Oncol.

[6] Jackson SP (2002). Sensing and repairing DNA double-strand breaks. Carcinogenesis.

[7] Helleday T (2003). Pathways for mitotic homologous recombination in mammalian cells. Mutat Res.

[8] Thacker J (2005). The RAD51 gene family, genetic instability and cancer. Cancer Lett.

[9] Hasselbach L, Haase S, Fischer D, Kolberg HC, Stürzbecher HW (2005). Characterisation of the promoter region of the human DNA-repair gene RAD51. Eur J Gynaecol Oncol.

[10] Antoniou AC, Sinilnikova OM, Simard J (2007). RAD51 135G-->C modifies breast cancer risk among BRCA2 mutation carriers: results from a combined analysis of 19 studies. Am J Hum Genet.

[11] Krivokuca AM, Malisic EJ, Dobricic JD, Brotto KV, Cavic MR, Jankovic RN, Tomasevic ZI, Brankovic-Magic MV (2014) RAD51 135G>C and TP53 Arg72Pro polymorphisms and susceptibility to breast cancer in Serbian women. Fam Cancer.13: 173–80 10.1007/s10689-013-9690-3 24114315

[12] Wang W, Li JL, He XF, Li AP, Cai YL, Xu N, Sun SM, Wu BY (2013). Association between the RAD51 135 G > C polymorphism and risk of cancer: a meta-analysis of 19,068 cases and 22,630 controls. PLoS ONE.

[13] Romanowicz-Makowska H, Smolarz B, Zadrozny M, Westfa B, Baszczyński J, Kokołaszwili G, Burzyfiski M, Połać I, Sporny S (2012). The association between polymorphisms of the RAD51-G135C, XRCC2-Arg188His and XRCC3-Thr241Met genes and clinico-pathologic features in breast cancer in Poland. Eur J Gynaecol Oncol.

[14] Romanowicz-Makowska H, Smolarz B, Zadrozny M, Westfal B, Baszczynski J, Polac I, Sporny S (2011). Single nucleotide polymorphisms in the homologous recombination repair genes and breast cancer risk in Polish women. Tohoku J Exp Med.

[15] Lee KM, Choi JY, Kang C, Kang CP, Park SK, Cho H, Cho DY, Yoo KY, Noh DY, Ahn SH, Park CG, Wei Q, Kang D (2005). Genetic polymorphisms of selected DNA repair genes, estrogen and progesterone receptor status, and breast cancer risk. Clin Cancer Res.

[16] Shin A, Lee KM, Ahn B, Park CG, Noh SK, Park DY, Ahn SH, Yoo KY, Kang D (2008). Genotype-phenotype relationship between DNA repair gene genetic polymorphisms and DNA repair capacity. Asian Pac J Cancer Prev.

[17] Kuschel B, Auranen A, McBride S (2002). Variants in DNA double-strand break repair genes and breast cancer susceptibility. Hum Mol Genet.

[18] Thacker J, Zdzienicka MZ (2004). The XRCC genes: expanding roles in DNA double-strand break repair. DNA Repair (Amst).

[19] Silva SN, Tomar M, Paulo C, Gomes BC, Azevedo AP, Teixeira V, Pina JE, Rueff J, Gaspar JF (2010). Breast cancer risk and common single nucleotide polymorphisms in homologous recombination DNA repair pathway genes XRCC2, XRCC3, NBS1 and RAD51. Cancer Epidemiol.

[20] Lin WY, Camp NJ, Cannon-Albright LA (2011). A role for XRCC2 gene polymorphisms in breast cancer risk and survival. J Med Genet.

[21] Sliwinski T, Krupa R, Majsterek I (2005). Polymorphisms of the BRCA2 and RAD51 genes in breast cancer. Breast Cancer Res Treat.

[22] Wang N, Xiu-juan D, Rong-miao Z, Wei G, Xiao-juan Z, Yan L (2009). An investigation on the polymorphisms of two DNA repair genes and susceptibility to ESCC and GCA of high-incidence region in northern China. Mol Biol Rep.

[23] Lu J, Wang LE, Xiong P, Sturgis EM, Spitz MR, Wei Q (2007). 172G > T variant in the 5′ untranslated region of DNA repair gene RAD51 reduces risk of squamous cell carcinoma of the head and neck and interacts with a P53 codon 72 variant. Carcinogenesis.

[24] Davidson JM, Gorringe KL, Chin SF, Orsetti B, Besret C, Courtay-Cahen C, Roberts I, Theillet C, Caldas C, Edwards PA (2000). Molecular cytogenetic analysis of breast cancer cell lines. Br J Cancer.

[25] Loveday RL, Greenman J, Simcox DL, Speirs V, Drew PJ, Monson JR, Kerin MJ (2000). Genetic changes in breast cancer detected by comparative genomic hybridisation. Int J Cancer.

[26] Zhao M, Chen P, Dong Y, Zhu X, Zhang X (2014). Relationship between Rad51 G135C and G172T variants and the susceptibility to cancer: a meta-analysis involving 54 case–control studies. PLoS ONE.

[27] Smolarz B, Zadrożny M, Duda-Szymańska J, Makowska M, Samulak D, Michalska MM, Mojs E, Bryś M, Forma E, Romanowicz-Makowska H (2013). RAD51 genotype and triple-negative breast cancer (TNBC) risk in Polish women. Pol J Pathol.

[28] Hosseini M, Houshmand M, Ebrahimi A (2013). RAD51 polymorphisms and breast cancer risk. Mol Biol Rep.

[29] Romanowicz-Makowska H, Smolarz B, Gajęcka M, Kiwerska K, Rydzanicz M, Kaczmarczyk D, Olszewski J, Szyfter K, Błasiak J, Morawiec-Sztandera A (2012). Polymorphism of the DNA repair genes RAD51 and XRCC2 in smoking- and drinking-related laryngeal cancer in a Polish population. Arch Med Sci.

[30] Improta G, Sgambato A, Bianchino G, Zupa A, Grieco V, La Torre G, Traficante A, Cittadini A (2008). Polymorphisms of the DNA repair genes XRCC1 and XRCC3 and risk of lung and colorectal cancer: a case–control study in a Southern Italian population. Anticancer Res.

[31] Lu PH, Li C, Zhou HY, Wang T, Chu CQ, Shen W, Tao GQ (2011). Same data sources, different pooled analysis result: the ongoing uncertainty in the subgroup analysis of RAD51 135G/C polymorphism and breast cancer risk. Breast Cancer Res Treat.

[32] Sliwinski T, Walczak A, Przybylowska K (2010). Polymorphisms of the XRCC3 C722T and the RAD51 G135C genes and the risk of head and neck cancer in a polish population. Exp Mol Pathol.

[33] Li F, Li C, Jiang Z, Ma N, Gao X (2011). XRCC3 T241M polymorphism and bladder cancer risk: a meta-analysis. Urology.

[34] Smolarz B, Samulak D, Michalska M, Góralczyk B, Szyłło K, Lewy J, Sporny S, Kokołaszwili G, Burzyński M, Romanowicz-Makowska H (2011). 135G > C and 172G > T polymorphism in the 5′ untranslated region of RAD51 and sporadic endometrial cancer risk in Polish women. Pol J Pathol.

